# Five‐Year Results of One‐Piece Zirconia Oral Implants Supporting Three‐Unit Fixed Dental Prostheses

**DOI:** 10.1111/clr.14407

**Published:** 2025-01-27

**Authors:** Ralf‐Joachim Kohal, Kirstin Vach, Frank Butz, Sebastian Berthold Maximilian Patzelt, Felix Burkhardt

**Affiliations:** ^1^ Medical Center ‐ University of Freiburg, Center for Dental Medicine, Department of Prosthetic Dentistry, Faculty of Medicine University of Freiburg Freiburg Germany; ^2^ Institute of Medical Biometry and Statistics Medical Center ‐ University of Freiburg Freiburg Germany

**Keywords:** clinical investigation, fixed dental prosthesis, oral implant, prospective, zirconia implant

## Abstract

**Objectives:**

The purpose of the present prospective case series was to investigate the clinical and radiological outcome of one‐piece zirconia implants fabricated from 3Y‐TZP with a moderately roughened endosseous surface (Sa = 1.24 μm) to support three‐unit fixed dental prostheses (FDP) after five years in function.

**Materials and Methods:**

Twenty‐seven patients received a total of 54 implants in a one‐stage surgery with immediate provisionalization. Peri‐implant bone loss was assessed using standardized radiographs. Additionally, soft‐tissue parameters were analyzed. Statistical analyses were conducted using linear mixed regression models and Wilcoxon Signed Rank tests (*p* < 0.05). All patients participated in an annual maintenance program.

**Results:**

Eighteen implants were lost up to the 5‐year follow‐up, resulting in a cumulative survival rate of 66.67%. The mean marginal bone loss of the remaining implants amounted to 1.89 mm. Probing depth, clinical attachment loss, and bleeding on probing increased from prosthesis insertion to the 5‐year follow‐up, while the plaque index showed no significant changes during the same period.

**Conclusions:**

The investigated one‐piece zirconia implant showed low survival rates as compared to previously reported survival rates of one‐piece zirconia or two‐piece titanium implants. Implant failure was attributed to peri‐implantitis, leading to the necessity of implant removal. The implant is not commercially available.

## 1 Introduction

Besides the gold standard titanium as an implant material, zirconium dioxide (zirconia; ZrO_2_) has emerged as a potential alternative material for oral implants (Morton et al. 2018; Pieralli et al. 2017; Roehling et al. 2023). The demand for zirconia oral implants has increased in recent years, as titanium particles and corrosive products could pose a potential health risk and may contribute to peri‐implantitis (Albrektsson et al. 2019; Fretwurst et al. 2018; Hosoki et al. 2016). Additionally, zirconia implants could positively influence the aesthetic result due to their tooth‐like color compared to grayish titanium implants (Cosgarea et al. 2015). Zirconia is biocompatible and demonstrates low plaque affinity, thereby exhibiting soft tissue‐friendly properties. It is expected that both titanium and zirconia implants will continue to be clinically applied in the future, with the demand for zirconia implants potentially increasing further (Sanz et al. 2019).

Zirconia oral implants are mostly made from 3 mol% yttria‐stabilized tetragonal zirconia polycrystal (3Y‐TZP). Numerous preclinical studies have shown a high bending moment, modulus of elasticity, and fracture toughness, mainly attributable to the mechanism of phase transformation toughening (Garvie, Hannink, and Pascoe 1975; Piconi and Maccauro 1999). Crack growth can be counteracted by phase transformation of adjacent zirconia grains, resulting in volume expansion and subsequent compression of the matrix (Christel et al. 1989). Micro‐rough surfaces have been found to facilitate improved osseointegration of zirconia implants, similar to titanium implants (Gahlert et al. 2012; Janner et al. 2018). In addition to commonly applied methods such as sandblasting, acid etching, and laser treatment (Altmann et al. 2017; Cattani‐Lorente et al. 2014), porous surfaces can be created using additive processes (Sennerby et al. 2005). Thus, zirconia implants are coated with a slurry of zirconia powder and a pore former before final sintering, leaving a porous surface after burning out the pore former (Adilstam and Iverhed 2003). In animal studies, implants with micro‐rough surfaces using this method showed significantly improved osseointegration compared to machined surfaces (Kohal et al. 2013a; Sennerby et al. 2005).

In recent years, the number of prospective clinical trials investigating zirconia oral implants as abutments for fixed restorations has increased (Brunello et al. 2022; Cionca, Hashim, and Mombelli 2021; Gahlert et al. 2022; Gargallo‐Albiol, Böhm, and Wang 2022; Koller et al. 2020; Steyer et al. 2021). However, these trials predominantly evaluate single crowns (SC) over a short period, reporting high short‐term survival rates. Prospective clinical studies on zirconia implants as abutments for fixed dental prostheses (FDP) are scarce (Balmer et al. 2020; Kohal et al. 2020; Lorenz et al. 2019). The initial short‐term results after one and three years showed an acceptable implant survival rate; however, inflammation‐induced bone loss increased significantly after three years, reducing the success rate of this system tremendously (Kohal et al. 2013b, 2023a, 2023b). However, there is no available data on the mid‐ and long‐term survival and success rates of this one‐piece zirconia implant system supporting a three‐unit FDP. Therefore, the objective of this prospective long‐term cohort study was to evaluate the clinical outcome of this zirconia oral implant with an additively coated porous surface to support a three‐unit FDP after five years. The aim of the present study was to clinically and radiologically re‐evaluate the one‐piece zirconia oral implants supporting three‐unit FDP.

## 2 Materials and Methods

### 2.1 Study Population

Prior to this prospective investigation, informed consent was obtained from all 27 patients participating in the study. Comprehensive inclusion and exclusion criteria were delineated in a precedent publication (Kohal et al. 2013b). The study protocol adhered to the STROBE statement guidelines for observational cohort studies and received approval from the ethics committee of the University Medical Center Freiburg, Freiburg, Germany (investigation number: 337/04).

### 2.2 Clinical Assessment

#### 2.2.1 Investigated Implant System

The one‐piece oral implants were fabricated from 3Y‐TZP (ZiUnite, Nobel Biocare, Gothenburg, Sweden) and featured a design similar to the NobelDirect titanium implant (Nobel Biocare). The implants contained a threaded, tapered endosseous body, a transmucosal cylindrical collar, and an integrated abutment cylinder. The endosseous implant body and transmucosal collar were moderately roughened (arithmetic mean height Sa = 1.24 μm) using the ZiUnite surface technology (Nobel Biocare). This process created a porous surface by sintering the implants with a zirconia slurry and burnable pore former, as described in detail by Sennerby et al. (2005). The surface of the abutment part, where the FDP is placed, exhibited a machined finish. These implants were produced in lengths of 10, 13, and 16 mm, with regular (RP Ø 4.3 mm) and wide platform diameters (WP Ø 5.0 mm). The investigated ZiUnite implant system received the appropriate regulatory clearance (CE marking) but was not available on the market yet.

#### 2.2.2 Implant Surgery and Prosthetic Delivery

Prior to surgery, a radiographic assessment of the prospective implant site was conducted using cone beam computed tomography (Newtom 3G, Newtom, Marburg, Germany). Surgical and prosthetic procedures are described in detail in a precedent publication (Kohal et al. 2013b). During surgery, bone quality and quantity were clinically evaluated according to the classification by Lekholm and Zarb (1985). Implants were placed either immediately after tooth extraction or in healed sites. For healed sites, two surgical approaches were used: a flapless technique with a tissue punch or a full‐thickness mucoperiosteal flap elevation. In the flapless method, a 2 mm twist drill, combined with a guided sleeve, was used to mark the implant's direction and position. A tissue punch guide was then inserted into the osteotomy, and a motorized punch removed the soft tissue at the implant site. The osteotomies were then expanded to the required dimensions before inserting the implants. For the flapped approach, a midcrestal incision, with or without vertical relieving incisions, was made, and the implants were placed following the manufacturer's instructions. Following implant placement, the abutment segment underwent minor preparation to place a temporary three‐unit prosthesis made of polymethyl methacrylate (PMMA). Occlusal and lateral contact points were removed to avoid any excessive forces on the implant during the healing phase. The final three‐unit FDPs were delivered for the mandible and maxilla after two or four months post‐implant placement, respectively. These FDPs comprised a zirconia framework (Procera) and a glass–ceramic veneering material (NobelRondo, both from Nobel Biocare), commonly used at the time of the commencement of the study. Cementation of all restorations was performed using a glass‐ionomer cement (Ketac Cem, 3M Espe, Neuss, Germany), with meticulous removal of any cement remnants.

#### 2.2.3 Follow‐Ups

Radiographic assessment was conducted utilizing standardized radiographs captured with the parallel technique and customized stents on the day of implant placement (baseline), at prosthetic delivery, and during the 1‐year, 3‐year, and 5‐year follow‐up appointments. Bone remodeling was assessed by calibrating the radiographs using the known width of the base of the implant abutment part, with the lower edge of the abutment serving as the reference point for measurements. Since the reference points varied in height between the mesial and distal regions of the implant due to the differing start of the threads, bone loss was evaluated mesially and distally, and an average value was calculated. All of the radiographs were independently assessed by a radiologist at the University of Gothenburg, Sweden. The clinical evaluations of both implants and adjacent teeth included pocket probing depths (PD), clinical attachment loss (CAL), bleeding on probing (BOP), and plaque index (PI). These clinical assessments were conducted at the time of prosthesis insertion and at the 1‐year, 3‐year, and 5‐year follow‐ups. Implant success criteria at 5 years were defined as follows: PD ≤ 5 mm, no bleeding on probing, no suppuration, and maximal bone loss of ≤ 2 mm (1 mm after 1 year after implant installation and 0.2 mm annually thereafter). The case definition for peri‐implantitis comprised positive bleeding on probing/suppuration, probing depth of ≥ 6 mm, and bone loss of > 2 mm (Berglundh et al. 2018; Karoussis et al. 2003; Östman et al. 2007; Schwarz et al. 2007). Patients were enrolled in an annual maintenance program that included regular check‐ups, oral hygiene instruction, and professional cleanings. Implant success criteria were adopted from van Steenberghe (van Steenberghe 1997).

### 2.3 Statistical Analysis

Descriptive statistics, including mean values and standard deviations, were reported for normally distributed data, while non‐normally distributed data were summarized using the median along with the 25% or 75% quartiles. The cumulative survival rates of zirconia implants were determined through actuarial life table analysis (Altman 1999). Imputation of missing bone level values at implant placement was performed utilizing data from subsequent time points and the mean changes observed across the entire study population. Linear mixed models were employed for the analysis of changes and subgroup differences in bone levels, with pairwise comparisons conducted using the Scheffé method to correct for multiple testing. Due to the non‐normal distribution of BOP and plaque index values, Wilcoxon Signed Rank tests were conducted on the patient level to evaluate differences between teeth and implants. The Kaplan–Meier survival rate was calculated for the implants. Cox models including the patient as a cluster were used for group comparisons. With the exception of BOP and PI, all analyses were performed at the implant level adjusted for age and gender in the corresponding regression models, including patient as a random effect. The significance level was set at 0.05. All statistical analyses were performed using STATA 17.0 (StataCorp, College Station, Texas, USA).

## 3 Results

The patients included in the present investigation lost their replaced teeth due to periodontitis (three patients) and due to caries (24 patients). The systemic health conditions of 22 patients were classified as good, with no medication intake reported. However, three patients were on antihypertensive medication, and two patients were receiving treatment for hypothyroidism. No serious illnesses were reported. None of the patients was a smoker, and none was identified as suffering from bruxism. A total of 54 implants were placed in 27 patients (11 females/16 males) to support 27 three‐unit FDPs. The age distribution at the time of the implant surgeries was as follows: one patient was between 18 and 20 years, three patients were between 31 and 40 years, five between 41 and 50 years, seven between 51 and 60 years, and 11 patients were between 61 and 70 years old, with a mean age of 53.4 ± 11.2 years. Forty‐four implants were inserted in the mandible (all in the posterior area) and 10 implants in the maxilla (four in the anterior area, six in the posterior area) (Table 1).

**TABLE 1:** Implant dimensions and survival after 5 years.

Platform [Ø mm]	Length [mm]	Maxilla		Mandible	
		Placed	Failed	Placed	Failed
RP 4.3	10	0	0	10	2
	13	4	1	10	1
	16	0	0	1	0
	**Total**	4	**1**	21	**3**
WP 5.0	10	1	0	12	6
	13	3	2	9	5
	16	2	1	2	0
	**Total**	6	**3**	23	**11**

Of the 54 implants, five implants were inserted immediately after tooth extraction, while 49 implants were placed into healed sites. Of these 49 implants, two implants were inserted using a flapless technique, five implants were placed with a punch of the soft tissues, and the remaining were placed in with a flap surgery. The majority of implant sites exhibited bone quantity of B and bone quality of II according to the criteria established by Lekholm and Zarb (1985). Furthermore, eleven implants displayed insertion torques below 35 Ncm, 28 implants ranged between 35 and 45 Ncm, and 15 implants exhibited insertion torques exceeding 45 Ncm.

### 3.1 Survival of the Fixed Dental Prostheses

Twenty‐six patients received 26 fixed dental prostheses (FDPs). At the 1‐year follow‐up, all 26 FDP were in situ. Eight of the FDP showed signs of chipping that could be polished (100% survival; 69% success). 24 patients with 24 FDPs were seen at the 3‐year follow‐up. 14 chippings were observed in total (100% survival; 42% success). At the 5‐year follow‐up, 15 patients with 15 FDPs were available for evaluation. Additional chip‐off fractures in the veneering ceramic did not occur, but one of the 15 FDPs required replacement due to severe chipping. This resulted in an FDP survival rate of 93.3% (29% success). No framework fractures or decementations were observed throughout the investigation.

### 3.2 Implant Survival

**FIGURE 1:**
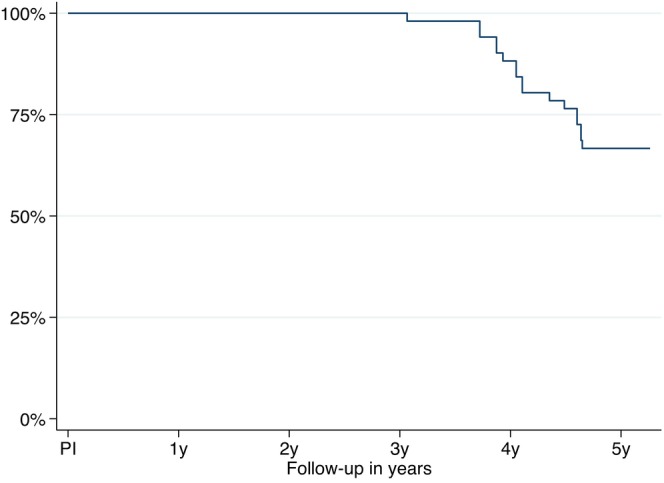
Kaplan–Meier survival estimate of the implants from implant placement (IP) to the 5‐year follow‐up (5 y) (IP = implant placement; PI = prosthesis insertion).

One implant failed during the healing period and was subsequently removed (WP = wide platform implant, length 16 mm), resulting in the exclusion of the patient from the study. Consequently, the final FDP was delivered to 26 of the 27 patients. Until the 3‐year follow‐up, one patient dropped out due to severe illness and was unavailable for further follow‐ups. Between the 3‐ and 5‐year follow‐ups, ten patients experienced the loss of 17 implants due to peri‐implant tissue destruction and were thus excluded from the study (Table 1; Figure 1). The remaining 15 patients completed the 5‐year follow‐up. The calculated implant survival rate was 66.67% [CI: 52.0%–77.78%].

Among the factors examined, only implant platform and bone quantity had a significant influence on implant survival. WP implants exhibited a significantly lower survival rate compared to implants with a regular platform (RP) (HR 3.4; *p = 0.028*). Additionally, implants placed into bone with quantity B demonstrated a higher survival rate compared to other bone quantities. Furthermore, subgroups were small and unevenly distributed (*p* = 0.005).

Owing to the limited sample size, it was not possible to assess the influence of the patient characteristic factors such as the reason for tooth loss, severe illnesses, ongoing medication, smoking, and bruxism on implant survival.

### 3.3 Marginal Bone Loss and Overall Implant Success

**FIGURE 2:**
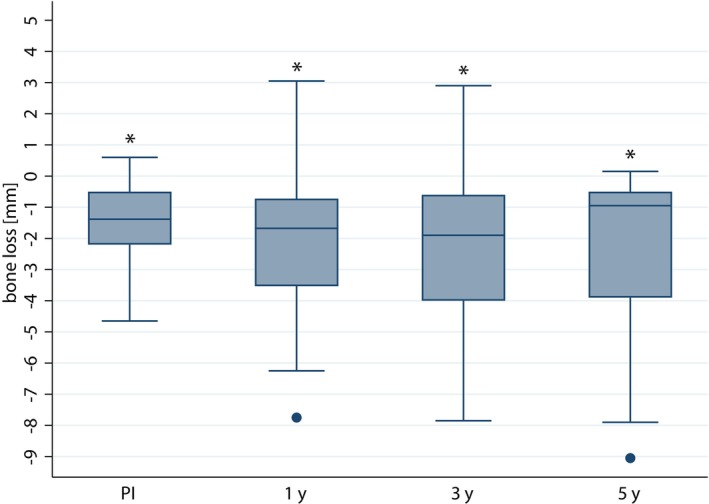
Boxplots showing bone loss (in mm) at the 1‐year (1 y), 3‐year (3 y), and 5‐year (5 y) follow‐ups in comparison to IP. Asterisks (*) indicate significances (*p* < 0.001) compared to IP.

At the 5‐year follow‐up, the standardized radiographs of the remaining implants revealed a bone loss of 1.89 ± 2.78 mm (Figure 2). This indicates a significant bone loss (difference 2.16 ± 2.46 mm, *p < 0.001*) compared to implant placement. In comparison, at the 1‐year follow‐up, the mean bone loss compared to baseline amounted to 2.13 ± 2.04 mm, and at the 3‐year follow‐up, it was 2.34 ± 2.54 mm. Standardized radiographs of 52 implants could be evaluated at the one‐year follow‐up, and radiographs of 44 implants were analyzable at the 3‐year follow‐up. However, only 27 implants could be assessed at the 5‐year follow‐up radiographically (the x‐rays of three implants could not be read).

At the 5‐year follow‐up, 1 implant gained bone (0.2 mm), 13 implants lost less than 1 mm, 2 implants lost between 1 and 2 mm, 3 implants lost between 2 and 3 mm, 3 lost between 3 and 4 mm, 2 implants lost between 4 and 5 mm, and 3 implants lost above 5 mm of bone. Eleven of the radiographically assessable 27 implants showed a marginal bone loss of more than 2 mm (41%). According to the (mere) success criteria for bone loss after 5 years (≤ 2 mm) (Karoussis et al. 2003), a 5‐year overall implant success rate of 59% was achieved.

A univariate analysis showed in all but one case no subgroup differences regarding the evaluated confounding factors in bone loss for the 5‐year follow‐up. Only the “no flap” design showed a significantly higher bone loss compared to the punch technique (*p = 0.018*). Nevertheless, it should be noted that the groups in this analysis were very small and not equally distributed. No other patient or implant‐related covariates (age, gender, jaw type, implant position anterior or posterior, bone quality, bone quantity, implant diameter, implant length, or bone grafting) showed an influence on bone loss.

### 3.4 Clinical Parameters

**FIGURE 3:**
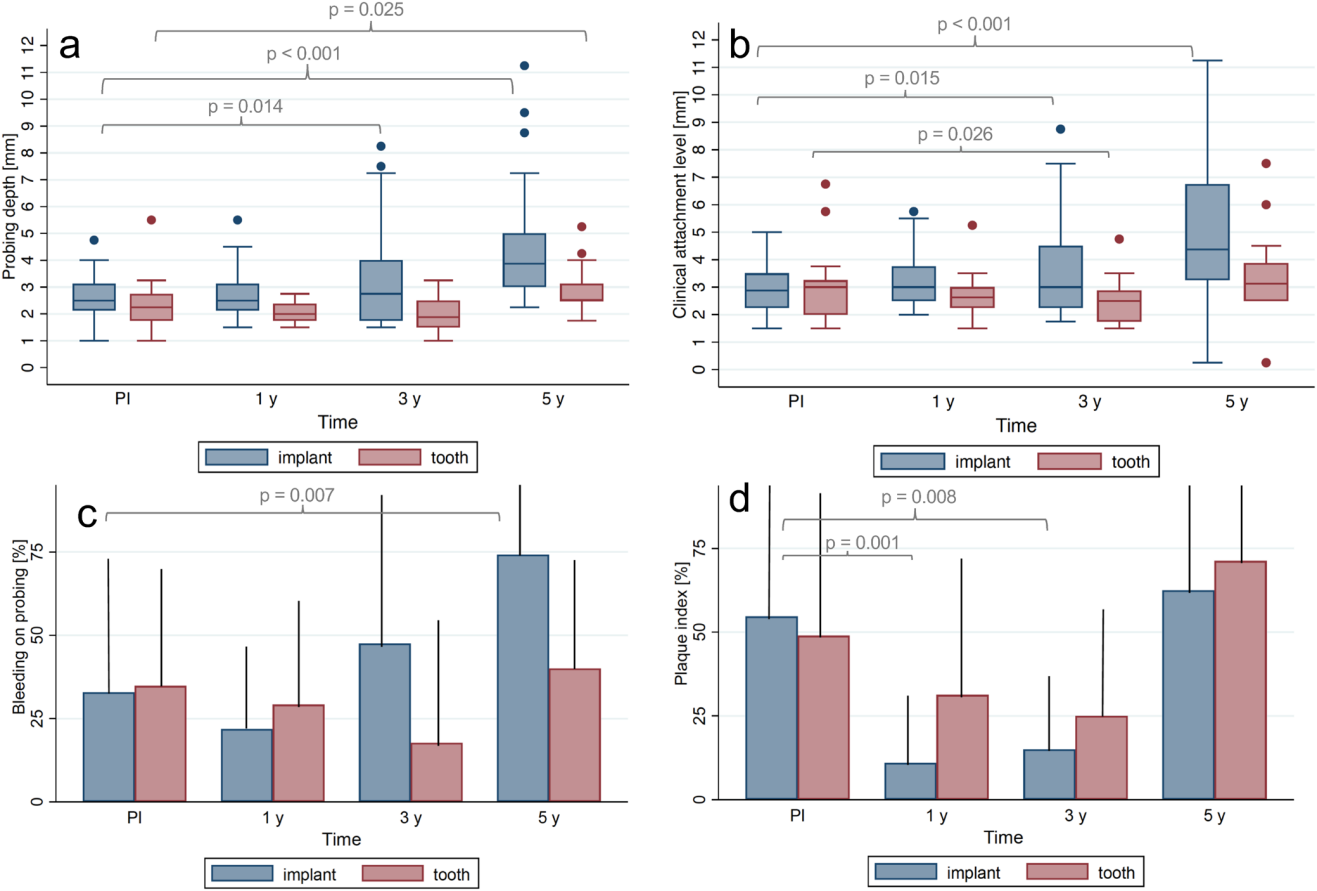
Probing depth (a), clinical attachment loss (b), bleeding on probing (c), and plaque index (d) at the prosthesis insertion (PI), 1‐year follow‐up (1 y), 3‐year follow‐up (3 y), and 5‐year follow‐up (5 y). Significant differences (*p* < 0.05) within each group (t, teeth; i, implants) are visualized compared to PI.

For the implants of the patients attending the 5‐year follow‐up, a probing depth (PD) of 4.48 ± 2.28 mm was measured (Figure 3a, Table 2). This indicated a significant increase compared to the PD at PI (2.64 ± 0.79 mm; *p < 0.001*), at the 1‐year follow‐up (2.76 ± 0.89 mm; *p < 0.001*), and at the 3‐year follow‐up (3.39 ± 1.98 mm; *p < 0.001*). An increase of PD from PI to the 5‐year follow‐up was observed for the reference teeth (*p = 0.025*) as well. However, the PD increased significantly more at the implant sites compared to the reference teeth (*p = 0.001*), independent of patients' gender and age. A frequency analysis of PD around teeth and implants revealed the following results: all but one tooth showed PD of 4 mm and less; one tooth showed a PD of 6 mm. Seven of the remaining 30 implants showed PD of 4 mm, six of 5 mm, four of 6 mm, one of 7 mm, two of 8 mm, two of 10 mm, and one of 12 mm on at least one implant side at the 5 year follow‐up.

**TABLE 2:** Soft‐tissue parameters: Probing depth (PD), clinical attachment loss (CAL), bleeding on probing (BOP), and plaque index (PI) after prosthesis insertion, at the 1 year‐, 3 year‐, and 5‐year follow‐ups, mean [standard deviation].

		PD [mm]	CAL [mm]	BOP [%]	PI [%]
Prosthesis insertion	Implants	2.64 (0.79)	2.98 (0.87)	32.81 (41.37)	54.69 (41.85)
	Reference teeth	2.33 (0.86)	3.00 (1.22)	34.78 (36.73)	48.91 (41.61)
1 y	Implants	2.76 (0.89)	3.21 (0.97)	21.86 (25.20)	10.94 (20.02)
	Reference teeth	2.05 (0.37)	2.69 (0.76)	29.17 (30.99)	31.25 (40.55)
3 y	Implants	3.39 (1.98)	3.73 (1.93)	47.50 (39.58)	15.00 (20.34)
	Reference teeth	2.07 (0.60)	2.50 (0.78)	17.70 (28.05)	25.00 (33.97)
5 y	Implants	4.48 (2.28)	5.07 (2.67)	74.17 (33.79)	62.50 (39.25)
	Reference teeth	2.90 (0.87)	3.25 (1.62)	40.00 (33.83)	71.25 (30.65)

At the 5‐year follow‐up, a clinical attachment loss (CAL) of 5.07 ± 2.67 mm was measured at the implant sites (Figure 3b, Table 2). This represents a significant increase (all *p < 0.001*) compared to PI (2.98 ± 0.87 mm), to the 1‐year follow‐up (3.21 ± 0.97 mm), and to the 3‐year follow‐up (3.73 ± 1.93 mm). The CAL at the implant sites did not change significantly between PI and the 1‐year follow‐up (*p = 0.24*), whereas significant changes were observed between the other time points (*p* < 0.05). Similar to PD, a significantly higher increase in CAL at the implant sites compared to the reference teeth was observed between PI and the 5‐year follow‐up (*p = 0.001*) independent of patients' age and gender.

At the 5‐year follow‐up, BOP of 74.17% ± 33.79% was measured at the implant sites (Figure 3c, Table 2). No significant change in BOP was observed between PI (32.81% ± 41.37%) and the 1‐year follow‐up (21.87% ± 25.20%), but it significantly increased by the 3‐year follow‐up (47.50% ± 39.58%) and again at the 5‐year follow‐up (both *p < 0.05*). This represents a significant change in BOP after 5 years compared to PI (*p < 0.01*), whereas the BOP at the reference teeth showed no significant alteration (*p = 0.41*). Therefore, after 3 and 5 years, a significantly higher BOP was observed at the implant sites compared to the reference teeth (both *p < 0.01*).

At prosthesis insertion, a plaque index of 54.69% ± 41.85% was measured, which showed a significant decrease at the 1‐year (10.94% ± 20.02%) and the 3‐year follow‐up (15.00% ± 20.34%), respectively (both *p < 0.02*) (Figure 3d, Table 2). However, the plaque index significantly increased again at the 5‐year follow‐up (*p < 0.002*), resulting in no significant differences compared to prosthesis insertion (*p = 0.54*). No significant differences were observed between reference teeth and implants at the respective time points (*p > 0.05*; 5‐year follow‐up: *p* = 0.17).

### 3.5 Peri‐Implant Conditions

Peri‐implant health, peri‐implant mucositis, and peri‐implantitis at the remaining implants were categorized according to Berglundh et al. (2018). Of the remaining 30 clinically evaluated implants, ten implants showed peri‐implant health (no BOP, no erythema, no swelling, no suppuration), seven showed peri‐implant mucositis (BOP positive, PD ≤ 5 mm, bone loss ≤ 2 mm), and 13 implants suffered from peri‐implantitis (BOP positive, suppuration positive, PD ≥ 6 mm, bone loss > 2 mm). Peri‐implant infections were treated before final removal following the C.I.S.T. protocol (Lang et al. 2004).

## 4 Discussion

The present study describes the 5‐year outcomes of a one‐piece zirconia oral implant supporting a three‐unit fixed dental prosthesis (FDP). A low cumulative implant survival rate of 66.67% was observed, since 17 implants had to be removed due to peri‐implantitis between the 3‐year and 5‐year follow‐up. Additionally, 20 of the remaining implants exhibited either signs of peri‐implant mucositis or peri‐implantitis. Given that titanium implants demonstrate significantly higher survival rates (95.6%–97.2%) and success rates after a 5‐year observation period, it has to be stated that the clinical result of the present implant system is unacceptable (Jung et al. 2008, 2012; Pjetursson et al. 2012). Although this implant system was not introduced to the market, the value of the present investigation lies in its mid‐ and long‐term examination following the initial observation of increased bone loss at the implant sites. Furthermore, zirconia implant system developments might benefit from the present insights.

In a recently published systematic review and meta‐analysis (Roehling et al. 2023), a mean survival rate of 97.2% for one‐ and two‐piece zirconia implants after 5 years was reported in contrast to the findings of the present study (66.67% survival rate). However, only six clinical cohort studies were included, as an inclusion criterion was the continued availability of the implant system on the market. Among these, only three studies investigated both SC and FDP (Balmer et al. 2020; Borgonovo et al. 2021; Kohal et al. 2020), without specifically evaluating the influence of the restoration type (FDP, SC) on implant survival. In another systematic review (Pieralli et al. 2017), an expected implant survival rate of > 95% after 5 years was calculated. Notably, there was no apparent influence on implant survival based on whether the implants were restored with SC or FDP. Further recent clinical investigations on zirconia implants restored with both SC and FDP also demonstrated high survival rates. For instance, Lorenz et al. (2019) observed a 100% survival rate for 83 zirconia implants after an extended observation period of 7.8 years. Similar to the present investigation, the evaluated implant restored with SC showed a low cumulative survival rate of 78.2% after 5 years for 66 implants (Kohal et al. 2023a). Hence, it can be inferred that the restoration type (FDP, SC) was not a determining factor for the observed lower implant survival rates of this implant system. The univariate analysis indicated a significantly higher loss of implants with a wide diameter platform compared to regular diameter platforms after 5 years. This was in contrast to most of the studies demonstrating no influence of the implant diameter on the survival rate with diameters between 4.0 and 5.5 mm (Gul et al. 2024). However, some earlier studies also reported a higher loss of wide‐diameter implants (Ivanoff et al. 1999; Renouard and Nisand 2006). A potential reason might be the use of wide‐diameter implants when primary stability had not been achieved with regular‐diameter implants or the presence of poor bone density. In the present study, the bone quantity of 2 according to Lekholm and Zarb (1985) showed higher survival rates than the other bone quantities. However, these results can only be seen as an orientation, as the subgroups were not large and varied in size.

The success and survival of oral implants are influenced by various patient characteristic factors such as the patient's age, reason for tooth loss (e.g., periodontitis, caries, trauma), and the overall condition of the oral environment. Older patients, especially those above 60, may face slightly higher risks of implant failure, primarily due to age‐related systemic conditions or reduced bone density. However, age alone is not considered a major influence for implant survival or success if the patient's overall health is managed well (Chrcanovic et al. 2018; De Baat 2000). In the present investigation, the systemic health conditions of the patients had to be rated as good. Three patients took only medication to reduce their high blood pressure, and two patients were using medication for hypothyroidism. Both conditions, obviously, do not influence implant survival/success (Hamadé, El‐Disoki, and Chrcanovic 2024; Torrejon‐Moya et al. 2022). All other patients did not take any medication. The reason for tooth loss may also further influence implant survival and success. Patients who lost teeth due to periodontitis may face higher implant failure rates, particularly if they are susceptible to further periodontal issues (Orishko et al. 2024). Effective periodontal therapy and maintenance are crucial for implant success in these patients. Three patients in the present investigation lost their teeth due to periodontitis. One of those patients lost both implants up to the 5‐year follow‐up. Whether tooth loss from caries poses a risk for implant success is not well evaluated, and a final conclusion can therefore not be drawn. Most of the patients of the present investigation lost their teeth due to caries. Implants replacing teeth lost due to trauma often have high success rates, assuming there is adequate bone structure and the surrounding tissues are healthy (Nørgaard Petersen, Jensen, and Dahl 2022). However, no patient in the present investigation lost teeth due to trauma. Systemic health conditions like diabetes, smoking, and the patient's commitment to maintaining good oral hygiene also play significant roles in the long‐term survival of implants. However, in the present investigation, the influence of diabetes and smoking could not be evaluated since none of the patients had these characteristics.

Similar to the 5‐year follow‐up of SC, the present study revealed a lower mean marginal bone loss compared to the 3‐year results (3y: −2.34 mm; 5y: −1.89 mm). However, it is important to note that between these two follow‐up assessments, 17 implants had to be removed due to massive bone loss caused by peri‐implant infection. The persistence of these implants in situ would have resulted in a significant increase in marginal bone loss at the 5‐year follow‐up. In the aforementioned systematic review and meta‐analysis (Roehling et al. 2023), a mean marginal bone loss of 1.1 mm was reported after 5 years, with the included studies demonstrating a high level of consistency. Despite the considerable number of implants removed due to bone loss, 11 of the in situ remaining implants (41%) exhibited a bone loss > 2 mm and 10 of these showed signs of peri‐implantitis. In similar investigations with zirconia implants exceeding survival rates of 94%, a maximum of 8% of the implants in situ showed a bone loss > 2 mm (Balmer et al. 2020; Grassi et al. 2015; Kohal et al. 2020). The univariate analysis, which evaluated potential factors on bone loss, can solely serve as an indicator, as it considers only the implants that remained in situ. In the three‐year analysis, when all implants were still in situ (except one early loss) but already exhibited advanced bone loss, no influence of confounding factors was observed (Kohal et al. 2023a). After 5 years, the surgical “no flap” technique showed a significantly higher bone loss compared to the punch technique, although these two groups were very small and the majority of the implants were placed with a flap approach. While flapless techniques represent minimally invasive procedures, the accurate placement of implants is hindered by limited visibility (Romandini et al. 2023). The application of different surgical techniques for implant placement represents a limitation of this study.

The 17 removed implants exhibited advanced marginal bone loss along with significantly increased PD and suppuration/pus, leading to the diagnosis of peri‐implantitis (Berglundh et al. 2018; Lang, Berglundh, et al. 2011; Schwarz et al. 2018). The remaining implants in situ demonstrated a progressive increase in PD over the years, reaching a mean peak value of 4.48 mm at the 5‐year follow‐up, accompanied by an elevated CAL of 5.07 mm. Increased PD and CAL can be observed at implant sites compared to teeth without a sign of peri‐implant infection (Gerber et al. 2009). However, after removal of 17 implants due to the peri‐implantitis (showing highly increased PD and CAL), the observed PD was still higher compared to ceramic implants in the described systematic review and meta‐analysis (PD of 3.0 mm after 5 years) (Roehling et al. 2023). Although radiographs showed less advanced bone loss in the remaining implants compared to earlier follow‐ups, the increased PD and CAL can be seen as indicators of ongoing inflammatory soft tissue responses around the implants. In addition, the elevated bleeding on probing (BOP) of 74%, which has continuously increased since the 1‐year examination, indicates another sign of inflammation. Although peri‐implant diseases are often plaque‐induced, no significant difference was observed regarding the plaque index after 5 years compared to prosthesis insertion and reference teeth, despite the high detected amount of plaque (63% at implant sites after 5 years). Given that the ability to clean the restoration can also influence the development of peri‐implant diseases (Schwarz et al. 2018), particular attention was paid to ensure that the FDP were not overcontoured and allowed for thorough cleaning of the pontic areas. The low plaque index at the 1‐ and 3‐year examinations confirmed the feasibility of meticulously cleaning the FDP restorations.

The micro‐rough ZiUnite surface of the investigated zirconia implant facilitated favorable osseointegration compared to machined surfaces, as demonstrated in animal studies using rabbit bone (Sennerby et al. 2005). This was confirmed by histological examinations of the removed zirconia implants, which showed a comparable osseointegration to titanium implants, indicating that a lack of osseointegration was not the cause of failure (Kohal et al. 2016). However, the micro‐rough surface of the implant body can also promote increased biofilm formation (Al‐Ahmad et al. 2013). As bone loss progresses, a greater extent of the micro‐rough surface area becomes exposed. In conjunction with a high prevalence of plaque accumulation (as observed in the 5‐year examination), this scenario may influence biofilm formation, which contributes to the advancement of peri‐implant tissue degeneration (Berglundh et al. 2018; Lang et al. 2011; Schwarz et al. 2018). Crystallographic analyses of explanted zirconia implants with this microrough surface also revealed an increased transformation of tetragonal to monoclinic zirconia grains (t‐m transformation) (Kohal et al. 2024; Sanon 2014). Due to the interconnection of the pores, water molecules could penetrate into the interior of the zirconia implant and accelerate the aging of the zirconia surface in the warm and humid environment (low temperature degradation) (Sanon et al. 2013). The exceptionally high degree of aging might have exerted stress on the bone by the increased volume of the zirconia grains caused by t‐m transformation (Chevalier et al. 2009). In addition, aging of the surface of these implants could result in a loss of the coating, which might have a negative effect on the bone‐implant interface and contribute to the poor results of this implant system.

Besides the applied technology for creating the micro‐rough surface, the implant's tapered design is unique. This design might have contributed to an increased pressure on the crestal part of the bone during implant placement, resulting in an elevated bone loss compared to other zirconia implant systems (Pieralli et al. 2017; Roehling et al. 2023). A comparable phenomenon was observed with the similar design of the NobelDirect titanium implant, which also exhibited increased bone loss in some clinical investigations (Albrektsson et al. 2007; Östman et al. 2007).

In summary, it can be assumed that the observed bone loss and peri‐implantitis may result from a combination of the aforementioned factors. Since the macro‐design of the implants and micro‐rough surface are distinctive compared to other zirconia implant systems, these factors may have played a major role in contributing to peri‐implant tissue degradation. One limitation of this study is the small number of patients and, consequently, small subgroups, thus limiting the reliability of the univariate analysis to provide definitive conclusions. The absence of a control group comprising another zirconia or titanium implant of a different design represents another limiting factor (Balmer et al. 2020).

Another potential limitation was the lack of standardized surgical techniques. Both flap procedures without a releasing incision and flapless surgeries using a punch were employed. Additionally, implants were sometimes placed immediately following tooth extraction, and in certain cases, minor bone grafting was carried out.

## 5 Conclusions

The investigated one‐piece zirconia implant exhibited much lower survival and success rates compared to both one‐piece zirconia and two‐piece titanium implants reported in previous studies. After the loss of one implant during the early healing phase before prosthetic insertion, 17 implants were lost due to peri‐implantitis up to the five‐year follow‐up. The remaining implants in situ exhibited increased bone loss and clinical signs of pathology, i.e., soft tissue edema, bleeding on probing, and suppuration. The therapeutic outcomes for this implant system were notably poor and very unsatisfactory, a result that could already be inferred from the 1‐ and 3‐year follow‐ups. Consequently, these implants were not made available to the market. However, the present results might help to advance the understanding of zirconia behavior in vivo.

## Author Contributions

**Ralf‐Joachim Kohal:** conceptualization, investigation, methodology, formal analysis, writing – review and editing, resources. **Kirstin Vach:** validation, software, formal analysis, writing – review and editing, visualization. **Frank Butz:** investigation, writing – review and editing. **Sebastian Berthold Maximilian Patzelt:** investigation, writing – review and editing. **Felix Burkhardt:** writing – original draft, formal analysis, visualization, validation.

## Conflicts of Interest

Ralf‐Joachim Kohal was a lecturer for Nobel Biocare from 2005 to 2008. Frank Butz was a lecturer for Nobel Biocare in 2007.

## Data Availability

The datasets generated and analyzed during the current study are available from the corresponding author on reasonable request.
